# Familial aggregation of primary open angle glaucoma in Shanghai, China

**Published:** 2013-08-27

**Authors:** Xiangmei Kong, Wenqing Zhu, Xueli Chen, Yuhong Chen, Xinghuai Sun

**Affiliations:** 1 Department of Ophthalmology and Vision Science, Eye and ENT Hospital of Fudan University, Shanghai, China; 2 Shanghai Key Laboratory of Visual Impairment and Restoration, Shanghai, China; 3 State Key Laboratory of Medical Neurobiology, Institutes of Brain Science, Fudan University, Shanghai, China

## Abstract

**Purpose:**

To identify familial aggregation of primary open angle glaucoma (POAG) in first-degree relatives in Shanghai, China.

**Methods:**

This was a prospective case-control study. First-degree relatives of 113 POAG patients and 119 normal controls underwent a standardized ophthalmic examination. Each participant was diagnosed as normal, glaucoma suspect or glaucoma. The prevalence of glaucoma and glaucoma suspect in each group was calculated, and the odds ratio (OR) and 95% confidence interval (CI) for family history were estimated using the Generalized Estimating Equations model.

**Results:**

Of 531 first-degree relatives in the case group, 67 (12.62%) were identified to have POAG, a rate eight times higher than that of the control group (8 of 526, 1.52%). In family units, the prevalence OR value of glaucoma was 8.77 (95% CI: 3.73–20.62). The effect of family history on parents, siblings, and offspring of probands was statistically significant, with OR values of 6.92 (95% CI: 1.90–25.18), 11.29 (95% CI: 3.63–35.11), and 11.35 (95% CI: 1.69–76.21), respectively. In the case of glaucoma suspect, a significant effect was found for both family units (OR 5.60; 95% CI: 1.15–27.21) and offspring (10.83 OR; 95% CI: 1.34–87.73).

**Conclusions:**

In Shanghai, relatives of glaucoma patients have a strongly increased risk of glaucoma, and priorities for glaucoma screening should target this population. The study demonstrates that familial aggregation of POAG transcends racial and cultural boundaries.

## Introduction

Glaucoma is the leading cause of irreversible blindness worldwide and affects about 70 million people, with Asians accounting for almost half of the world’s glaucoma patients [1,2]. Primary open angle glaucoma (POAG) affects 0.71–2.1% of the population in China [3-5]. Given the large population and expected future growth of China, this disease poses a great burden on patients, the society, and the health economy. POAG tends to progress slowly, and patients are often asymptomatic until the disease is in its advanced stages. Early diagnosis is beneficial in controlling the progression of glaucoma and improving prognosis. Regular ophthalmic examination plays a key role in early diagnosis and treatment, particularly for patients with known risk factors.

According to the Preferred Practice Pattern guidelines of the American Academy of Ophthalmology [6], likelihood of developing glaucomatous optic neuropathy increases with the following risk factors: elevated intraocular pressure (IOP), older age, family history of glaucoma, thinner central corneal thickness, African or Hispanic/Latino descent. A known family history of glaucoma is an important risk factor, providing a simple method to systematically screen patients at potential high risk for glaucoma and help increase the detection rate of the disease. In the Rotterdam Study, the risk ratio for glaucoma was 9.2 (95% confidence interval (CI): 1.2–73.9) [7]; in the Baltimore Eye Study, the odds ratio (OR) was 2.85 (95% CI: 1.82–4.46) [8]. Furthermore, in the Barbados Eye Study, researchers found a fourfold increase in the risk of developing glaucoma in siblings of known glaucoma patients [9]. However, these studies primarily involved patients of Caucasian and African descent, and to the best of our knowledge, no large studies have examined the risk of glaucoma among Chinese patients with a known family history of the disease.

In a previous study conducted in Shanghai, we reported that 21.49% of POAG patients had a family history of glaucoma, and the OR for POAG was 8.38 (95% CI: 3.33–21.07) [10]. A limitation of that study, however, was that family history information was obtained by patient recall. As such, inaccuracies of recall or misclassification could have distorted our findings. In the present study, standardized ophthalmological examinations of the first-degree relatives of patients were obtained. The aim of this study was to determine the familial aggregation of patients with POAG in Shanghai, China.

## Methods

### Study population

We performed a prospective case-control family study from October 2010 to April 2012 at the Eye and Ear, Nose, Throat Hospital (EENT) of Fudan University in Shanghai. EENT is one of the top eye hospitals in China, seeing patients mainly from Shanghai and nearby provinces of east China. The study examined both proband and screening subjects. The proband subjects were enrolled in the study first, and then their first-degree relatives were enrolled as screening subjects. The inclusion criteria for case probands were Chinese descent, adult age (20 years or older), and POAG diagnosis before enrollment. POAG was diagnosed based on glaucomatous disc cupping and reproducible visual field damage, open angle, excluding other secondary causes, in one or both eyes. A total of 113 case probands were included in this study. The inclusion criteria for control probands were Chinese descent and absence of glaucoma, and the control population was age- and gender-matched to the case probands. The control probands came from a pool of volunteers recruited by a poster displayed in the hospital, many of whom were cataract patients or residents in the nearby community. A total of 119 control probands were enrolled. The mean age of the probands was 58.53±13.70 in case group and 57.82±13.82 in control group. The number of males was 57 in case group and 56 in control group. All the first-degree relatives (parents, siblings, and offspring) of case probands and control probands were invited to be screening subjects and complete a comprehensive ophthalmic examination to determine their glaucoma status. It was expected that some screening subjects might not accept examination because of death, residence abroad, or other reasons. Response rates were calculated. Generally 531 relatives in case group and 526 in control group accepted the examination. The mean age of the relatives was 54.94±16.29 in case group and 55.28±16.06 in control group. The number of males was 265 in case group and 229 in control group.

Written informed consent was obtained from each participant. The project was approved by the medical ethics committee of EENT and conducted in accordance with the revised Declaration of Helsinki.

### Examination protocol

All participants accepted a comprehensive ophthalmic examination. Uncorrected visual acuity was measured (E charts) at a distance of 5 m. Automatic refractometry (Auto Refractometer AR-610; Nidek Co, Ltd., Tokyo, Japan) was performed if uncorrected visual acuity was lower than 1.0. Slit-lamp examinations were performed by an experienced ophthalmologist who was blind to the status of each group. If any participant had narrow-angle glaucoma, gonioscopy was performed with a Goldmann one-mirror lens (Haag Streit, Bern, Switzerland) at 25X magnification with low ambient illumination. Digital photographs of the optic nerve and macula were taken using a fundus camera (CR-DGi non-mydriatic retinal camera; Canon Inc, Kyoto, Japan). The vertical cup-to-disc ratio (VCDR), cup notch, narrowed optic disc rim, optic disc margin hemorrhage, and retinal nerve fiber layer defect were used as indicators of structural glaucomatous change. The grading process used VCDR from 0.1 to 1.0 in 0.1 increments. IOP was measured using a Goldmann applanation tonometer (Haag Streit AG, Bern, Switzerland). If the VCDR was greater than 0.3, the thicknesses of the retinal nerve fiber layer around the optic disc and the ganglion cell complex in the macular area were measured by RTVue Optical Coherence Tomography (Optovue, Inc., Fremont, CA), and 24–2 Humphrey visual field examinations (Humphrey Field Analyzer Model 750; Zeiss Humphrey Systems, Dublin, CA) were obtained. Tests were considered reliable and eligible for analysis if there were fewer than 33% false-positive, 33% false-negative, and 20% fixation losses. Demographic information, including gender, age, and relationship to the probands, was also collected.

### Definition of glaucoma and glaucoma suspect

Glaucoma was defined according to the criteria set forth by the International Society for Geographical and Epidemiological Ophthalmology [11]. A category 1 diagnosis (structural and functional evidence of glaucoma) was defined as a VCDR or an intereye asymmetry in the VCDR at or exceeding the 97.5th percentile for the normal population, or a neuroretinal rim width reduced to ≤0.1 VCDR (between 11 and 1 o’clock or 5 and 7 o’clock), in addition to a definite visual field defect consistent with glaucoma. A category 2 diagnosis (advanced structural damage with unproven visual field loss) was characterized by a VCDR or VCDR asymmetry at or exceeding the 99.5th percentile for the normal population. A category 3 diagnosis (for eyes in which the optic nerve head could not be examined or for which a visual field examination was not possible) was a visual acuity <3/60 combined with either an intraocular pressure exceeding the 99.5th percentile or definite glaucoma medical records, such as filtering surgery history. Because our study focused on POAG, we excluded those subjects with an occludable drainage angle (where the posterior, usually pigmented trabecular meshwork was not visible for 270° or more during a static examination) and those with an identifiable secondary cause.

Glaucoma suspect was also defined according to the criteria of the International Society for Geographical and Epidemiological Ophthalmology [11]. Those who met category 1 (but not category 2) disc criteria but were not proven to have definite field defects were labeled as disc suspect, while those who had definite field defects but did not meet category 1 disc criteria were considered field suspect. In addition, those with optic disc margin hemorrhage or IOP equal to or exceeding the 97.5th percentile were also included as glaucoma suspect. After all the examinations, an experienced ophthalmologist who was blind to the participants’ status in the case group or control group classified each participant as normal, glaucoma suspect or glaucoma.

### Statistical analysis

All analyses were performed using a commercially available statistical software package (SPSS for Windows, version 15.0; SPSS, Chicago, IL). Data on means were presented as mean ± SD. The prevalence of glaucoma was calculated and compared for both groups. Prevalence figures were adjusted for age and gender. As the first-degree relatives for each case or control patient may share similar genetic or environmental conditions, the usual independence assumption for a case-control study would not hold for a proband study. Logistic regression analysis was thus inappropriate. Instead, we chose the Generalized Estimating Equations model, which accounted for the correlation within each group of first-degree relatives to estimate the prevalence OR of glaucoma. OR and 95% confidence intervals were presented. All p values were two-sided and were considered statistically significant when the values were less than 0.05.

## Results

### General information

All participants in the study were of Chinese Han origin. The age and gender of the 113 case probands and 119 control probands were comparable (Table 1).

**Table 1:** General information of the probands in both groups.

Variable	Control group	Case group	P value
Number	119	113	
**Age**			
Mean(s.d.),year	57.82 (13.82)	58.53 (13.70)	0.987
Range	26–87	20–87	
**Gender**			
Male,n(%)	56(47.06)	57(50.44)	0.761

Totally 119 control probands and 113 case probands with primary open angle glaucoma were recruited in this study. The age and gender in both groups were comparable.

The response rates of first-degree relatives in the case group and the control group were 82.3% (531 subjects) and 81.2% (526 subjects), respectively. The median VCDR with normal visual fields was 0.4, with 97.5th and 99.5th percentiles of 0.7 and 0.8, respectively. The mean and the 97.5th and 99.5th percentiles for absolute difference in VCDR between both eyes were 0, 0.2, and 0.3, respectively. For IOP, the mean and 97.5th and 99.5th percentiles were 15 mmHg, 21 mmHg, and 25 mmHg, respectively. These figures were used as cutoff values for categories in the diagnostic definitions of glaucoma and glaucoma suspect [11]. The participants’ age, gender and relationship to proband are shown in Table 2. The prevalence of glaucoma among first-degree relatives of case probands was 8.3 times higher than in the control group (12.62% versus 1.52%). The prevalence of glaucoma suspect was 5.6 times higher in the case group than in the control group (3.20% versus 0.57%).

**Table 2:** General information of the relatives accepted glaucoma screening in both groups

Variable	Control group	Case group	P value
Number	526	531	
**Age of relatives, mean(s.d.), range**			
Total relatives	55.28(16.06), 6–85	54.94(16.29), 5–88	0.995
Parents	70.89(8.89), 51–85	70.37(11.34), 44–88	0.816
Siblings	59.53(9.89), 27–84	59.87(9.76), 13–88	0.693
Offspring	37.68(12.18), 6–66	36.90(11.82), 5–61	0.565
**Gender, n(%)**			
Male	229(43.54)	265(49.91)	0.536
**Relationship to probands, n(%)**			
Parents	103(19.58)	94(17.70)	0.431
Siblings	267(50.76)	280(52.73)	0.578
Offspring	156(29.66)	157(29.57)	0.955
**Status of disease, n(%)**			
Normal	515(97.91)	447(84.18)	0.028
Glaucoma suspect	3(0.57)	17(3.20)	0.000
Glaucoma	8(1.52)	67(12.62)	0.000

The numbers of the relatives accepted screening were 526 in control group and 531 in case group. The age, gender, and relationship to the probands were comparable. While the percentage of glaucoma and glaucoma suspect were much higher in case group than in control group (12.62% versus 1.52%, 3.20% versus 0.57% respectively).

### Odds ratio for glaucoma by family unit

**Figure 1:**
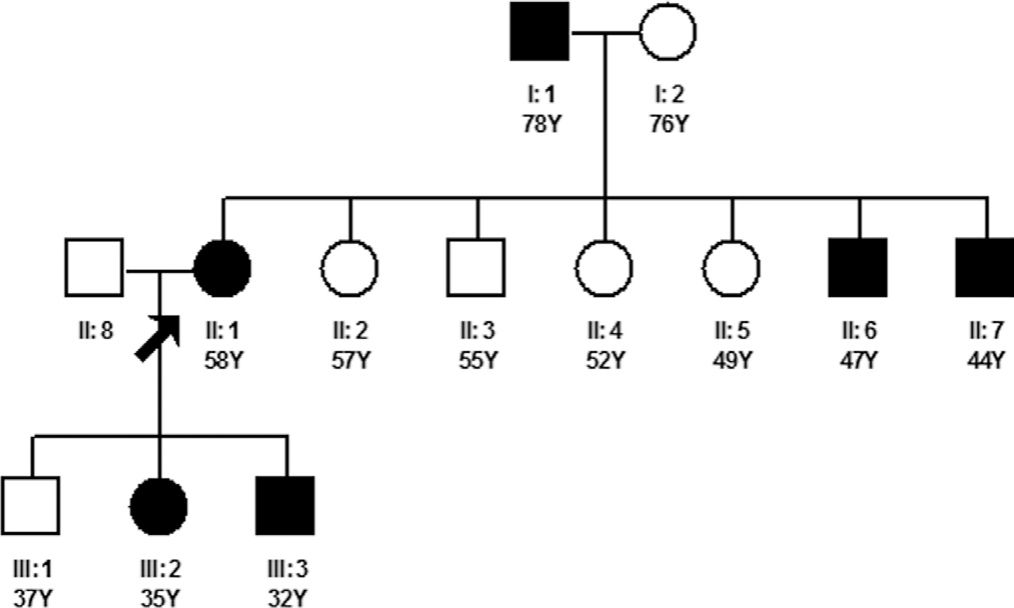
A typical pedigree in the case group. The proband, a 58-year-old female diagnosed with primary open angle glaucoma, had 11 first-degree relatives. Five of these relatives—her father, two younger brothers, one daughter and one son—were also glaucoma patients.

In this study, we defined “family history” as having a first-degree relative diagnosed with POAG. To investigate the effect of family history on all members in a family, we chose to use family units as the study object. A typical pedigree is shown in Figure 1; this family unit includes 12 members in three generations, of whom six (the proband and five relatives) have glaucoma. Family units with no family history of glaucoma refer to the 119 normal controls, and family units with a family history of glaucoma refer to the 113 case groups. The OR value for glaucoma in family units with a family history of the disease was 8.77 (95% CI: 3.73–20.62, p<0.0001), and the value for glaucoma suspect was 6.85 (95% CI: 1.95–24.06, p=0.018); see Table 3.

**Table 3:** Odds ratio (OR) and 95% confidence interval (CI) for glaucoma and glaucoma suspect in family units

Variable	Glaucoma			Glaucoma Suspect		
	No	Yes	P value	No	Yes	P value
**Family history**						
No	112	7		116	3	
Yes	73	40		96	17	
OR (95%CI)	8.77(3.73–20.62)		<0.0001	6.85(1.95–24.06)		0.018

Family units without family history of glaucoma refer to 119 normal controls, and family units with family history of glaucoma refer to 113 case groups. The OR value for a family history for glaucoma in family units was 8.77 (95%CI:3.73–20.62) and 6.85 (95%CI:1.95–24.06) for glaucoma suspect.

### Odds ratio for glaucoma by relationship to the proband

First-degree relatives are parents, siblings, and offspring. The numbers of parents, siblings, and offspring diagnosed as normal, glaucoma suspect, or glaucoma were calculated in both groups, and the effects of family history on different relatives of the probands were analyzed.

More screening subjects were diagnosed with glaucoma in the case group than in the control group. After adjusting for age and gender, the effect of family history was statistically significant for parents, siblings, and offspring of POAG patients, with OR values of 6.92 (95% CI: 1.90–25.18, p=0.003), 11.29 (95% CI: 3.63–35.11, p<0.0001), and 11.35 (95% CI: 1.69–76.21, p=0.012), respectively; see Table 4.

**Table 4:** Odds ratio (OR) and 95% confidence interval (CI) for glaucoma in different relationships to the probands

Variable	Control group	Case group	OR (95% CI)	P value
**Parents of probands**				
Normal	99	76	6.92 (1.90–25.18)	0.003
Glaucoma	3	15		
**Siblings of probands**				
Normal	262	235	11.29 (3.63–35.11)	<0.0001
Glaucoma	4	40		
**Offspring of probands**				
Normal	154	136	11.35 (1.69–76.21)	0.012
Glaucoma	1	12		

After adjusted with age and gender, the effect of family history on parents, siblings and offspring were all statistically significant, with OR values of 6.92 (95% CI:1.90–25.18), 11.29 (95% CI:3.63–35.11) and 11.35 (95% CI:1.69–76.21), respectively.

Among individuals diagnosed with glaucoma suspect, the effect of family history on offspring of POAG patients was of statistical significance, with an OR value of 10.83 (95% CI: 1.34–87.73, p=0.026) adjusting for age and gender. No statistical difference was found in parents (OR 3.93; 95% CI: 0.41–37.35, p=0.234) and siblings (OR 6.06; 95% CI: 0.70–52.68, p=0.103); see Table 5.

**Table 5:** Odds ratio (OR) and 95% confidence interval (CI) for glaucoma suspect in different relationships to the probands

Variable	Control group	Case group	OR (95% CI)	P value
**Parents of probands**				
Normal	99	76	3.93 (0.41–37.35)	0.234
Glaucoma suspect	1	3		
**Siblings of probands**				
Normal	262	235	6.06 (0.70–52.68)	0.103
Glaucoma suspect	1	5		
**Offspring of probands**				
Normal	154	136	10.83 (1.34–87.73)	0.026
Glaucoma suspect	1	9		

For glaucoma suspect, the effect of family history on offspring was of statistically significance, with OR value of 10.83 (95% CI:1.34–87373) adjusted with age and gender, while the effects on parents and siblings showed no statistical difference [OR 3.93 (95%CI:0.41–37.35) and OR 6.06 (95%CI:0.70–52.68)].

## Discussion

This study demonstrates a 12.62% prevalence of glaucoma and a 3.2% prevalence of glaucoma suspect among first-degree relatives of POAG patients. The risk of developing glaucoma was found to be much greater for these individuals than for those who have no family history of the disease. It has been previously reported that a family history of glaucoma puts an individual at greater risk of developing open angle glaucoma [12,13] and that first-degree relatives have a stronger positive correlation than extended family members [10,14]. The prevalence of glaucoma among siblings was reported to be 10.4% (6 of 61) in the Rotterdam study [7], 9.9% (16 of 161) in the Baltimore Eye Survey [8], and 11.8% (32 of 271), in the Nottingham study [15]. In the present study, we found that the prevalence of POAG in the sibling group was 14.5% (40 of 275), which was higher than in the aforementioned three studies, but less than in the Barbados Family Study, which found a prevalence of 19.8% (67 of 338) among siblings of Afro-Caribbean descent [16]. The differences in prevalence may be explained by the varying ethnic backgrounds of patients enrolled in the studies. For instance, it is well known that there is a higher prevalence of POAG among the black population (7%) than among whites (1.1–3%) [16-20], while the prevalence of POAG in Asians has been reported to be 0.71–2.1% [3-5]. Few studies about family history and POAG have focused on the Chinese population. In one population-based survey conducted in Harbin in northeast China [5], researchers demonstrated that the OR value of family history of glaucoma for POAG was 14.58 (95% CI: 6.05–35.15, p<0.001). A study conducted in Hong Kong [21] reported an OR value of 20.2 (95% CI: 2.18–187, p=0.008). However, these studies did not clearly define family history or specify the exact relationships between participants and the probands with glaucoma. Inclusion of other kinds of glaucoma and relatives of different degrees might result in overestimation of risk. Given the limited data regarding the prevalence of POAG among first-degree relatives in China, the present study provides important epidemiological data regarding the Chinese population. Furthermore, we did not rely on oral reports of glaucoma history, but rather employed a more stringent diagnostic definition of glaucoma for both probands and relatives, potentially helping to reduce the risk of misclassification.

Prior studies have noted a stronger association of POAG with a history of glaucoma in siblings than in parents or offspring. Similar environmental exposures among siblings may explain this. However, our study identified a significant percentage of parents and offspring of POAG patients who also had glaucoma. The prevalence of POAG in parents (16.5%; OR 6.92) and in offspring (8.1%; OR 11.35) of patients was much higher than in the Baltimore Eye Survey, which found a prevalence of 5.6% among parents and 1.2% among offspring [8]. Compared with families in western countries, Chinese parents and their children tend to reside in the same household for longer periods of time, and common environmental exposures may account for the difference in prevalence in the present study. Furthermore, the older age of patients in this study compared with our previous study may be related to the difference in prevalence of POAG in parents and offspring.

Glaucoma suspect has become an independent diagnosis and should be followed carefully. The prevalence of glaucoma suspect in case group offspring, 6.2% (9/145), was significantly higher than in the control group. Previous studies have shown an incidence of 0.1–0.6% per year [20,22,23], while Sung et al. reported a 1%-per-year incidence in siblings with a positive family history [15]. Although the prevalence of POAG increases with age [24], close attention should be focused on young patients. It is of utmost importance to target this high-risk group and develop a screening program that can identify glaucoma suspect. In particular, there is a need to increase awareness of the disease among offspring of glaucoma patients.

In the present study, we evaluated the accuracy of oral reports of glaucoma history among relatives for comparison with our previous study [10]. In the previous study, among 228 POAG patients, 49 (21.49%) reported a family history of glaucoma, and the OR was 8.38 (95% CI: 3.33–21.07). Except parents, siblings and offspring account for the family history rate of POAG [OR 8.99 (95% CI: 2.38–33.99) and OR 19.23 (95% CI: 1.53–241.24), respectively] [10]. Because the previous study relied on oral reports instead of screening of all the patient’s first-degree relatives, it could not provide information regarding the prevalence of glaucoma. In this study, the OR of family history for glaucoma was 8.77 in family units (95% CI: 3.73–20.62) and 6.92 (95% CI: 1.90–25.18), 11.29 (95% CI: 3.63–35.11), and 11.35 (95% CI: 1.69–76.21) in parents, siblings, and offspring, respectively. The trends were similar, but the absolute value was greater in the present study. This discrepancy may be explained by the larger sample size in this study and possible problems with recall associated with the prior study.

The main advantages of this study were as follows: (1) We did not rely on oral history alone but physically examined all first-degree relatives; (2) we used a standard grading system for glaucoma; and (3) ascertainment of probands and relatives was high and was similar in both case and control groups. Because of the limitation of sample size, we acknowledge that the large confidence intervals might have some effect on the estimated impact of a family history of glaucoma on a person’s relative risk of POAG. Also, the study was clinic-based rather than population-based, which might cause selection bias. However, the comparability of probands and relatives in the case and control groups should mitigate some of this bias.

In conclusion, we demonstrated that family history plays an important role in the prevalence of glaucoma in first-degree relatives in Shanghai, China. Familial aggregation of POAG transcends racial and cultural boundaries. Priorities regarding glaucoma screening should target first-degree relatives of POAG patients, especially those offspring who are relatively young and can expect to live a long time.
